# Surgery

**Published:** 1891-05

**Authors:** John Parmenter


### SURGERY.

Conducted by JOHN PARMENTER, M. D.

SCHMELTZ (*Gaz. Méd. de Strasbourg*, 1890, No. 7,) relates a case in which he amputated a breast during hypnotic anesthesia. The patient was twenty years of age, and suffered from a sarcoma of the right breast. From the beginning to the end, the operation was conducted as ordinarily; the face was very pale; the pulse showed scarcely any disturbance; union by first intention. Recovery in fourteen days. The parents had told the patient the hour of the operation, in consequence of which, on this day, complete anesthesia could not be obtained, hypnosis having been easily induced previously. The operation was, therefore, postponed until the following day, the patient having been informed that it would not take place for eight days. She was then hypnotized and the operation proceeded with.

---

Wagner (*Centralblatt f. Chirurg.*, 1891, No. 2,) describes two cases in which he has used his method of "temporary resection of the cranium," first described in No. 47 of the above for 1889. Both cases, given somewhat in detail, demonstrate that the operation is very easy to do, and that the resected piece reunites both quickly and without reaction. In both instances the part resected was about one-fifth of the entire cranial vault, which leads the author to believe that a still greater amount will heal in the same way, and he would not fear to resect two-fifths or more if necessary. Naturally the isthmus of the flap must be correspondingly broader. Just as we have trephined, sometimes, the skull in different places, so we can make this temporary resection in one or more places. First, corresponding to the piece of bone to be removed, a curved incision is made whose ends converge toward each other. This flap must overlap the bone to be raised one-fifth to two-fifths of an inch. The soft parts retract, and the incision gapes in a sickle-shaped way, with the point toward the base of the flap. The flap is now firmly pressed upon the cranial surface, and the pericranium smoothly cut through along its border. With a small, fine chisel a little trough is made in the bone along the line of the incision. For the further steps of the operation, a moderately thick but small chisel, abruptly beveled on one side, is used. This is applied in an oblique manner. One can work his way through the cranium most quickly with this. When all the bone is chiselled through, a small, thin chisel is used to cut through that part of the bone covered by the isthmus of the flap. The skin and bone are now raised together with the aid of a small elevator. The replacing later on is very easy to effect. When the chisel is applied obliquely, the inner circumference is naturally less than the outer. The soft parts are then sewed together with fine sutures, and one of the under angles of the wound left open. The suture line does not correspond with the bony defect, but is somewhat removed from it, the soft parts overlapping this, in accordance with the plan of the incision above mentioned. In this way an adherent scar between bone and skin is avoided.

Regarding the indications for "temporary resection," or "osteoplastic trepanation," the author regards it, in general, indicated wherever in not injured bone the cranial vault is to be opened, not only in cases where we expect with certainty or probability to find removable alterations, but where we only suspect them; for instance, many cases of epilepsy of doubtful traumatic origin. This form of exploratory opening of the cranium the author regards fully as justifiable and no more dangerous than exploratory laparatomy.

---

C. L. Dana (*Post-Graduate*, January, 1891,) gives a new method of determining the position of the fissure of Rolando. It is this: Find and mark the stephanion, *i. e.*, the point where the temporal ridge crosses the coronal suture. Find and mark the concave depression just above and behind the tip of the mastoid, and just below the asterion or junction of the lambdoid and temporo-parietal sutures. Draw a line between these points. Find the bregmia, and draw a line from it to the posterior edge of the external auditory meatus. *The point of crossing will be just over the lower end of the fissure of Rolando or within a centimetre of it.*

---

Wyeth (*Medical Record*, February 21, 1891,) describes a case of craniectomy for microcephalus. The history of the case is as follows:

Male child born at term, February 2, 1890. Normal labor, without deformity; weighed nearly six pounds; at four weeks of age the anterior fontanelle closed. At the third month child had whooping cough, became nervous, screamed as if in pain, and was extremely wakeful. In July chloral and morphia were given. September 27th, child had a cataleptic attack which lasted one and one-half hours, it remaining "as if dead" during this interval. Following this attack talipes equino-varus was noted, the left foot being most affected. The left hand was also somewhat flexed. November 21st child much improved, opiates discontinued and improvement in flesh and weight. No expansion, however, of cranium. January 1st, child came under Dr. Wyeth's care, looking fat and rosy. Cranium about as large as that of a child two months old and sharp pointed. Eyes moved convulsively at times, pupils dilated; did not seem to appreciate things about it. Hands and arms did not move coördinately to grasp things offered, but were chiefly engaged in jerky movements toward mouth. The various portions of the body, feet excepted, seemed normal. Operation January 7, 1891, chloroform, head shaved, antiseptic details. Incision in median line from near base of nose to just beyond occipital protuberance. Pericranium pulled aside to expose skull for about one inch on either side of median line; cranial bones firmly ossified. No interosseous cartilage observed. Small trephine applied, and two trenches made with rongeur, one-quarter inch wide and extending from just above the eyes to the occipital protuberance, leaving a bridge three-fourths of an inch wide to protect the superior ongitudinal sinus in its entire length. At either end two trenches were then cut—laterally one inch and the parietal bones divided with bone scissors one inch and a half in the middle of the top of the head in a direction toward the ear on either side. Four fingers were inserted beneath each half of the exposed skull bones and they were torn loose from the dura mater. This widened the trenches to one inch each. Dura not opened. Catgut ligatures, catgut wisps between skull and dura for drainage. Time, one hour and thirty minutes. Wound dressed on tenth day; union by first intention. Improvement followed from the first. One month after operation the deformities in the extremities had entirely disappeared and there was evidently a marked increase in intelligence. It noticed those about it, took hold of objects offered it, laughed and behaved like a child of six or eight months. Pupils normal and child otherwise greatly improved.

---

HALSTED (*Bulletin of the Johns Hopkins' Hospital*, Vol. II., No. X.,) gives here the substance of a demonstration of intestinal anastomosis, made before the Johns Hopkins' Hospital Medical Society. Three dogs, killed after an operation for intestinal anastomosis, showed in two instances no adhesions; in the other, a delicate adhesion at one spot between omentum and line of suture. The writer says: "The success of any form of intestinal suture is inversely proportionate to the extent of the adhesions which result from the employment of the particular methods." His method is new, and depends for its success upon the appreciation of the importance of the submucous coat of the intestine. Inasmuch as the existence of this coat seems to have been ignored, the writer was led to make experiments, of which this demonstration was a result. He then explained the nature of this coat, showed its strength, and how to recognize it by the increased resistance which it offers to the needle, showing at the same time how feeble is the hold which the suture has when it includes only serous and muscular coats of the intestine. For performing intestinal suture of any kind, the following statements are emphasized:

1. It is bad surgery to employ a stitch which enters the lumen of the intestine.
2. It is impossible to suture the serosa alone.
3. It is impossible to suture unfailingly the serosa and muscularis alone, unless one is familiar with the resistance offered to the needle by the coats of the intestine. Furthermore, stitches which include nothing but these two coats, tear out easily, and are, therefore, not to be trusted.

4. Each stitch should include a bit of the submucosa; a thread of this coat is much stronger than a shred of the entire thickness of the serosa and muscularis. It is not difficult to familiarize one's self with the resistance furnished by the submucosa, and it is quite as easy to include a bit of this coat in each stitch as to suture the serosa and the muscularis alone.

5. As many as possible of the stitches should be taken, and of these as many as convenient should be tied before the intestine is opened.

6. The quilt or square stitches are to be preferred to the Lembert's stitches, because one row of them (the former) is sufficient, and because they tear out less easily and constrict the tissues less than do the Lembert's stitches. Madelung's cartilage plates for circular suture of the intestine are superfluous when the square stitches are used, and when a bit of the submucosa is taken up with each stitch.

He then follows with a description of his method of putting in the sutures, which, however, requires the aid of his diagrams to make it easily comprehensible to any but a surgeon familiar with this kind of work. He claims for his method the following advantages:

1. None of the stitches perforate the intestinal wall.

2. All of the stitches are applied and more than half of them are tied before the intestine is opened.

3. The square stitches are employed.

A paragraph or two upon the preparation and preservation of the needles concludes an article well worth the perusal of the surgeon.

